# Graham’s Element of Chemistry

**Published:** 1852-09

**Authors:** 


*Graham's Elements of Chemistry.*

We are indebted to Messrs. Blanchard & Lea for part first of a new edition of Graham's Elements of Chemistry just issued. The present is a progressive age, and especially so in the departments of natural science. Every working student helps to create a demand either for new books, or for new editions of those already existing. The work has been for some time before the public, and has won for the author a deserved reputation. This new edition is a faithful compend of the science up to the present time. We wait anxiously for the second part.

It is for sale by Keen & Brothers, Chicago. J.

